# Beyond behavior: a neurocognitive agenda for investigating the neural underpinnings of successful high-technology augmentative and alternative communication in adults

**DOI:** 10.3389/fnhum.2026.1847242

**Published:** 2026-08-05

**Authors:** Szu-Han Kay Chen, Marisa Nagano, Laura M. Gonnerman, Hsinyi Tanya Liu, Erin L. Meier

**Affiliations:** 1 Department of Communication Sciences and Disorders, University of New Hampshire, Durham, NH, United States; 2 Department of Physical Therapy, University of South Dakota, Vermillion, SD, United States; 3 Department of Communication Sciences and Disorders, Northeastern University, Boston, MA, United States

**Keywords:** AAC, aphasia, fNIRS, spoken language, treatment

## Abstract

**Purpose:**

Research has shown that augmentative and alternative communication (AAC) has the potential to function not only as a compensatory communicative strategy, but also as a tool to facilitate language recovery for people with aphasia. However, much work remains in order to understand the brain changes associated with AAC-driven recovery. The purpose of this article is to highlight the need for neurological research on high-technology AAC use, and to advance that research agenda by proposing the Transformation of Representation Across Neurocognitive Systems for Concept to External Navigation and Display (TRANSCEND) framework, a testable model of language production for people with aphasia who use AAC. We also argue for fNIRS as a promising neuroimaging tool that offers the advantage of allowing ecologically valid AAC use during experimental tasks.

**Conclusion:**

The TRANSCEND framework, a dual-route neurological model that begins with concept retrieval and leads to either verbal or AAC output, provides a foundation for future research that teases apart the neural underpinnings of motor, speech, and language aspects of AAC use.

## Introduction

Augmentative and alternative communication (AAC) encompasses a broad array of tools and strategies designed to support individuals with significant communication impairments and enable them to participate more fully in daily life, education, and social interaction. For example, low-technology AAC strategies may include picture boards or the use of gestures, while high-technology systems include devices that output speech when users tap an icon on a tablet or operate a switch. It is estimated that at least 1% of the total population could benefit from AAC (Beukelman and Light, 2020). AAC has been widely used with children with autism, cerebral palsy, and other developmental conditions that limit natural speech. For these populations, it serves as a critical pathway to expressive communication and supports both language and speech development (Alzrayer et al., 2021; Holyfield et al., 2017; Pope et al., 2025; Pousada García et al., 2011; Schlosser and Koul, 2015).

Importantly, AAC is not limited to developmental conditions; it also plays a vital role in supporting individuals with acquired and degenerative communication disorders (Fried-Oken et al., 2006, 2012; May et al., 2024). Post-stroke aphasia provides a critical model for examining AAC use in acquired adult communication disorders because it disrupts core language processes while preserving the potential for recovery, making it possible to evaluate AAC as both a compensatory tool and a mechanism for supporting language rehabilitation. Despite the persistent myth that AAC use hinders verbal language use (Dietz et al., 2020), research has shown that AAC intervention actually facilitates recovery of language and communication in adults with post-stroke aphasia (Chen et al., 2026a; Chen et al., 2026b; Dietz et al., 2018; Huang et al., 2025), including by strengthening underlying linguistic processes (Dietz et al., 2020; Kraat, 1990; Weissling and Prentice, 2010). Several studies have demonstrated that AAC interventions can lead to improvements in overall language function in people with aphasia whether using low-technology (Huang et al., 2025) or high-technology AAC systems (Alam et al., 2023). These findings challenge the notion of “learned non-use,” a phenomenon in which individuals with aphasia may reduce or abandon attempts to use spoken language due to repeated communication failure and limited opportunities for recovery (Pulvermüller and Berthier, 2008). Rather than reinforcing “learned non-use,” AAC interventions that actively scaffold language production may help counteract this pattern by re-engaging individuals with the language system. By providing multimodal representations such as symbols or pictures paired with speech output and/or written words, AAC may facilitate repeated and supported language activation, thereby promoting neural reorganization and experience-dependent neuroplastic change.

To date, however, this potential for AAC to drive recovery remains underexplored, as relevant research has focused primarily on behavioral outcomes, with relatively little work examining the neural systems that support AAC-mediated communication. The small body of existing literature focuses on high-tech AAC and provides preliminary evidence that high-tech AAC use engages and may modify distributed neural systems involved in language, visual processing, and motor learning (Dietz et al., 2018; Dietz et al., 2014; Wilkinson et al., 2015a; Wilkinson et al., 2015b). For example, Dietz et al. (2018) found that individuals with aphasia who received high-tech AAC intervention demonstrated greater behavioral improvements in spoken language than those in a non-AAC treatment group, with fMRI evidence of a distinct leftward shift in activation and increased recruitment of visual processing regions among AAC treatment responders. Complementary fMRI evidence from adults without aphasia suggests that experience with AAC-like display grids engages neural systems involved in visual processing, memory, and motor learning during target selection tasks (Wilkinson et al., 2015a). However, this literature remains limited in both scope and ecological validity. Existing neuroimaging studies have largely relied on constrained tasks that do not capture the real-time demands of naturalistic high-tech AAC use. Moreover, without a neurocognitive model of high-tech AAC language production, it remains difficult to generate testable hypotheses about which neural systems should be engaged, how AAC-related neural activity should change with intervention, or what activation patterns constitute an optimal endpoint of neuroplastic change in AAC users.

## Motivation for an integrated neurocognitive model of verbal and high-technology AAC-mediated communication

High-tech AAC is designed to aid expression, and its use engages a broad set of cognitive and motor processes that partially overlap with those required for verbal production, including visuospatial encoding and integration, language, cognitive control, and motor planning and execution. Consider the processes involved in using a high-technology AAC system. First, the user must formulate the conceptual message that they wish to communicate. Next, they must visually perceive and comprehend the symbols, pictures, or written words on the AAC device display, before deciding which symbol, picture, or written word/phrase matches the conceptual message that they wish to convey, disregarding all non-target options. Finally, they must select the intended target on the display, creating and executing a motor plan with precise control, whether through fingers, hands, head, or eye gaze. If the individual wishes to construct longer or novel utterances, they must also navigate between multiple AAC displays, spell words, and assemble the components of spontaneous messages in the correct order (engaging the morphosyntactic system).

Critically, high-tech AAC-mediated language production does not occur in isolation from other expressive modalities. The same user may also attempt to produce the intended target verbally, either before, during, or after AAC-mediated output. This multimodal form of production raises a central unanswered question: how do AAC-mediated and spoken output interact in real time? Spoken production draws heavily on conceptual preparation, lexical-semantic retrieval, phonological and syntactic encoding, and articulation, whereas high-tech AAC use additionally engages the perceptual, visuospatial, selection, and device-directed motor processes described above. Multimodal AAC-speech production may therefore strengthen shared language processes, but it may also impose additional demands on cognitive control as users coordinate between partially overlapping output systems. This issue is especially important for adults with post-stroke aphasia who are learning high-tech AAC systems for the first time after the onset of language impairment. However, the field lacks direct neuroimaging comparisons of AAC-mediated, spoken, and combined production, leaving it unclear when multimodal output is facilitative, compensatory, or competitive. Although these component processes have been studied extensively in isolation, we contend that a fuller understanding of AAC’s capacity to promote neuroplastic change requires investigation of real-time AAC use, both on its own and when integrated with spoken output.

Moreover, current theoretical models offer important insights into component processes, but none fully captures the demands of high-tech AAC-mediated language production or its coordination with spoken output. For example, psycholinguistic models explain how intended meanings become spoken words (Dell and O’Seaghdha, 1992; Levelt et al., 1999), but they do not specify how lexical representations are mapped onto AAC displays or visual-symbolic selections. While motor control theories explain processes of speech articulation and/or goal-directed movement (Bohland et al., 2010; Scott, 2004; Shadmehr and Krakauer, 2008; Tourville and Guenther, 2011; Wolpert and Kawato, 1998), they do not integrate speech motor output with AAC-mediated motor output. Executive control models explain how users maintain goals, select among competing alternatives, inhibit non-targets, and coordinate multiple systems (Braver et al., 2021; Cieslik et al., 2015), but they do not specify the linguistic and AAC-specific representations being controlled. Together, these models provide complementary accounts of individual component processes but remain fragmented, leaving unanswered how linguistic, cognitive, and sensorimotor mechanisms interact during high-tech AAC-mediated language production and learning. Thus, a unified neurocognitive model that integrates these component process models is needed to generate testable predictions about the shared and distinct mechanisms supporting each modality and the conditions under which AAC may support spoken-language recovery. However, no such model currently exists.

To address this gap, we propose a testable neurocognitive model of high-tech AAC-based language production, the TRANSCEND Model (Transformation of Representation Across Neurocognitive Systems for Concept to External Navigation and Display), to guide future empirical research examining the behavioral and neural correlates of successful high-tech AAC use. This initial version of TRANSCEND focuses on single-word production as a starting point for building a foundational body of neurological research. In the sections that follow, we describe the TRANSCEND model in detail, before introducing functional near-infrared spectroscopy (fNIRS) as a promising and underutilized neuroimaging tool for studying real-time high-tech AAC use, across language, motor/visuospatial, and executive function domains as laid out in our model.

## TRANSCEND model

Because no single existing theory adequately explains the neurocognitive processes underlying high-tech AAC-mediated language production, the TRANSCEND model integrates complementary theoretical frameworks that collectively account for the major component processes involved in high-tech AAC spoken language production. Rather than providing an exhaustive review of all available theories, we selected well-established theoretical frameworks that have substantially advanced understanding of psycholinguistic processing (e.g., Dell and O’Seaghdha, 1992; Levelt et al., 1999), speech and motor control (e.g., Bohland et al., 2010; Scott, 2004; Tourville and Guenther, 2011), executive control (e.g., Braver et al., 2021; Cieslik et al., 2015), and motor learning (e.g., Shadmehr and Krakauer, 2008; Wolpert and Kawato, 1998). These frameworks were selected because each addresses a distinct component of language production, cognitive control, motor planning, or learning that is relevant to high-tech AAC-mediated spoken language production. Together, they provide complementary perspectives that inform the development of an integrated neurocognitive framework.

**Figure 1:**
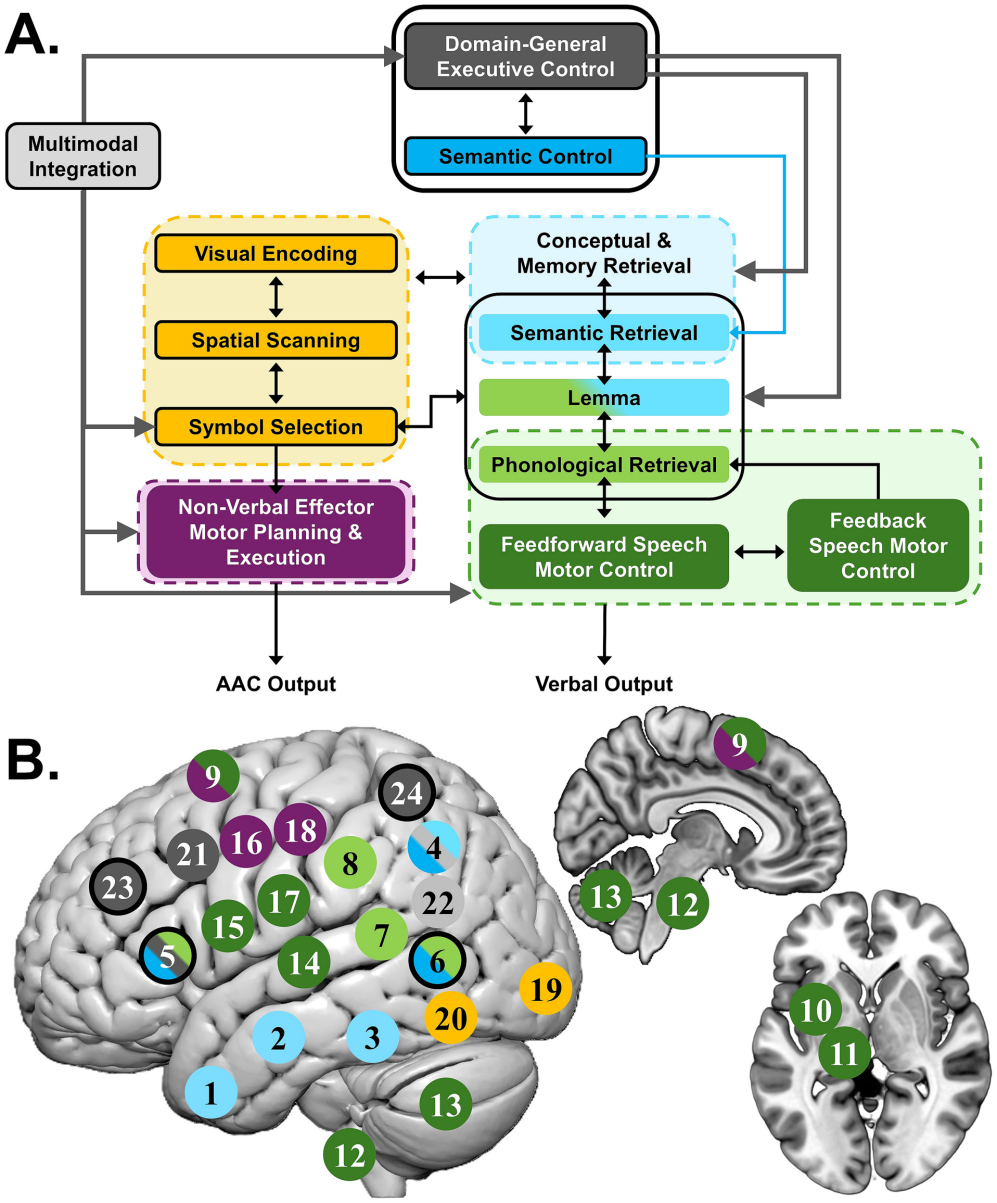
**(A)** TRANSCEND Model schematic, highlighting functional components and their connections. **(B)** Regions associated with the TRANSCEND model, color coded to correspond with the functional components illustrated in **(A)**. 1, anterior temporal lobe (ATL); 2, mid middle temporal gyrus (mMTG); 3, posterior inferior temporal gyrus (pITG); 4, angular gyrus (AG); 5, inferior frontal gyrus (IFG); 6, posterior middle temporal gyrus (pMTG); 7, posterior superior temporal gyrus (pSTG); 8, supramarginal gyrus (SMG); 9, supplementary motor area (SMA); 10, basal ganglia (putamen and globus pallidus); 11, thalamus; 12, pons; 13, cerebellum; 14, primary auditory area (A1); 15, primary motor area – face (M1f); 16, primary motor area – hand (M1h); 17, primary somatosensory area – face (S1f); 18, primary somatosensory area – hand (S1h); 19, primary visual area (V1); 20, visual word form area (VWFA); 21, frontal eye field (FEF); 22, temporoparietal junction (TPJ); 23, dorsolateral prefrontal cortex (DLPFC); 24, superior parietal lobule (SPL). Medial temporal regions implicated in memory are not pictured.

In light of the preceding discussion, we propose the TRANSCEND Model (Transformation of Representation Across Neurocognitive Systems for Concept to External Navigation and Display), illustrated in Figure 1A, which specifies a dynamic architecture composed of interacting neurocognitive components and dual interactive transformation routes through which internal conceptual representations are externalized. Certain systems are recruited regardless of output modality (e.g., domain general control and conceptual representation systems), but for AAC users, processing may proceed along two parallel but interacting routes: a verbal route supporting spoken production and an AAC route supporting symbol-based expression through a high-technology AAC system. These routes are structurally parallel, each involving representational activation and preparation for external output, yet they differ in the nature of the representational code and transformation demands required for expression. Importantly, the two routes may operate interactively rather than independently, allowing activation within one pathway to influence processing within the other. Figure 1B depicts the brain structures associated with each component of the model.

As a starting point, this version of the TRANSCEND model centers on single-word production because it offers a constrained context for modeling high-tech AAC use. Relative to phrase- or sentence-level production, single-word production reduces demands on syntax, discourse planning, and message-level integration, allowing the model to focus on core processes involved in conceptual preparation, lexical access, symbol/word selection, visuospatial search, and motor execution. As an integrated neurocognitive model of multimodal single-word production, TRANSCEND links psycholinguistic accounts of lexical access with motor-control accounts of output execution and neurocognitive theories of domain-general control to specify how an intended message is transformed into AAC-mediated, spoken, or combined output. As such, the model and its components draw heavily on the serial model of lexical retrieval by Levelt et al. (1999), Dell et al.’ interactive activation two-step model of lexical access (Dell et al., 1997; Dell and O’Seaghdha, 1992; Schwartz et al., 2006), as well as Tourville and Guenther (2011)‘s Directions into Velocities of Articulators (DIVA) model of speech motor control and the updated Gradient Order DIVA (GODIVA) model (Bohland et al., 2010). With a starting point of conceptual preparation for both routes, the model also incorporates theories regarding the neural organization of the semantic system and the potential role of semantic control for different output modalities (Binder and Desai, 2011; Mirman et al., 2017; Lambon Ralph et al., 2017). Finally, the backbone of the model is formed by theories of cognitive control such as the integrative theory of prefrontal cortex function by Miller and Cohen (2001) as well as the more recent Dual Mechanisms of Cognitive Control framework by Braver et al. (2021), and work regarding the dissociation between the language network and multiple demand system by Duncan (2010) and Fedorenko et al. (2024). Of note, these frameworks are not reworked wholesale within TRANSCEND; rather, their core principles are recontextualized for high-technology AAC use to specify the core processes outlined above and explained in greater detail below.

To illustrate how the TRANSCEND model operates, consider an individual who is describing a birthday party that they attended over the weekend. They intend to communicate the word “gift” to their communication partner, which they can do via verbal output and/or using a high-technology AAC system.

### Core components

#### Cross-cutting regulatory component: domain-general cognitive control

Rather than constituting a discrete stage of production, domain-general cognitive control is conceptualized in TRANSCEND as a cross-cutting regulatory system that supports goal-directed communication across AAC-mediated and spoken output. Contemporary cognitive neuroscience identifies a frontoparietal multiple-demand (MD) network—encompassing lateral frontal, dorsomedial frontal/insula, and superior parietal regions—as a core system for domain-general control that is recruited across diverse tasks as cognitive demands increase (Duncan, 2010; Fedorenko et al., 2011, 2013). Task encoding within the MD network is consistent with the distinction between subnetworks implicated in top-down control (Crittenden et al., 2016), including the fronto-parietal subcomponent that initiates and modulates control and the cingulo-opercular subnetwork that maintains stable set parameters over a task (Dosenbach et al., 2007, 2008). The MD network also encompasses the salience network, which mediates the detection of goal-relevant versus irrelevant stimuli and coordinates neural states (Menon and Uddin, 2010; Uddin, 2015). Thus, within TRANSCEND, this system supports the maintenance of communicative goals, allocation of attention, selection among competing representations, inhibition of non-target responses, task or modality shifting, and monitoring of action outcomes. These control demands are relevant to both spoken production and AAC use but are expected to be especially pronounced during high-technology AAC interaction given that in AAC, the communicative intention must be sustained across multiple transformation stages, including concept preparation, visual-symbol mapping, display navigation, target selection, and device-directed motor execution. In contrast to spoken language, high-tech AAC requires navigating an external interface, resolving competing symbol representations, maintaining spatial orientation within the display, and sequencing selections across pages, which additionally tax cognitive control. Furthermore, work by Fedorenko et al. indicates that the MD network is not highly engaged during language processing when task demands remain low and are linguistic in nature (Diachek et al., 2020; Fedorenko et al., 2011, 2024; MacGregor et al., 2022), a distinction we return to in the section.

#### Conceptual memory component

When an individual intends to produce the word “gift,” processing begins at the conceptual level for both spoken production and AAC-mediated output. The individual may retrieve an episodic memory associated with giving or receiving a gift, such as a recent birthday or holiday event; medial temporal structures and medial prefrontal regions support episodic and autobiographical memory retrieval. Simultaneously, semantic knowledge is activated. This includes world knowledge associated with the concept of a gift, such as its physical properties (e.g., has a bow, wrapped in paper), associations (e.g., holiday, celebration, recipient, balloons), and associated actions (e.g., is given). The semantic system has been described as a distributed network, either with the anterior temporal lobe functioning as a central amodal hub as proposed in the hub and spoke model (Lambon Ralph et al., 2017), multiple regions (angular gyrus, middle temporal gyrus, and posterior inferior temporal regions) serving as convergence zones (Binder and Desai, 2011), or via a dual-hub system, with the anterior temporal lobe and angular gyrus, respectively, responsible for mediating taxonomic and thematic relationships (Mirman et al., 2017). Regardless of the specific semantic framework, activation within this network results in a coherent conceptual representation corresponding to the intended meaning of “gift.” When competing semantic alternatives such as “balloon,” “toy,” or “cake” are also activated, controlled access to the target concept may require engagement of a semantic control network involving anterior portions of the left inferior frontal gyrus and posterior middle temporal regions (Hodgson et al., 2021; Lambon Ralph et al., 2017; Noonan et al., 2013). This controlled retrieval supports selection of the intended semantic representation before lexical encoding proceeds.

### Verbal route

#### Word form retrieval

Following retrieval of the conceptual representation in the Conceptual Memory Component, processing proceeds to lexical retrieval, which is often modeled as a two-step process (Dell and O’Seaghdha, 1992; Schwartz et al., 2006). At this stage, in certain lexical access models (Levelt et al., 1999), activation transitions from semantic representation to the lemma level, an abstract intermediary layer that links conceptual meaning to lexical form. The lemma represents the lexical item independent of its phonological structure and mediates between semantic activation and phonological encoding. For the intended concept of “gift,” the corresponding lemma is activated.

If semantically related alternatives such as “balloon” are co-activated, competition may arise at the lexical selection stage. Resolution of competing lexical candidates has been associated with posterior portions of the left inferior frontal gyrus, particularly pars triangularis, and posterior middle temporal regions, as key semantic control hubs that are responsible for controlled lexical selection and suppression of competing lexical candidates (Lambon Ralph et al., 2017). In the second stage, phonological codes corresponding to the word “gift” are retrieved and assembled through engagement of posterior superior temporal regions and inferior parietal areas, including the supramarginal gyrus, which are implicated in phonological processing and phonological working memory. This stage involves mapping the abstract lexical representation onto its phonemes and organizing them into a coherent phonological sequence prior to articulatory planning. Inferior frontal regions may further contribute to phonological sequencing and selection, particularly when competing phonological alternatives must be resolved.

#### Speech motor planning and execution

Following phonological assembly, the verbal route proceeds to speech motor planning and execution. Within the TRANSCEND model, this stage is grounded in the DIVA and GODIVA models of speech motor control, which specify the neural mechanisms of speech motor control, emphasizing how learned speech sounds are translated into articulatory motor commands through feedforward and feedback control systems (Bohland et al., 2010; Tourville and Guenther, 2011). Once a stable phonological representation of “gift” has been assembled, speech sound representations in the left ventral premotor cortex and inferior frontal regions activate learned motor programs that drive articulatory movements via the primary motor cortex and related motor pathways. Initiation of a learned speech motor program is regulated by an initiation map within the supplementary motor area (SMA)/pre-SMA, and basal ganglia-thalamo-cortical loops interact with the initiation map to support the appropriate timing, selection, and sequencing of speech motor programs. Cortico-cerebellar circuitry, including pontine relays between cortical motor regions and the cerebellum, supports the prediction, timing, and refinement of speech motor commands through feedforward learning and error-based updating. Simultaneously, auditory and somatosensory feedback systems monitor speech output. Auditory cortex supports comparison between predicted and actual acoustic signals, while somatosensory regions monitor articulatory positioning and movement. Discrepancies between predicted and actual feedback may trigger corrective adjustments through feedback control circuits involving posterior superior temporal regions and inferior parietal cortex. These feedback mechanisms allow ongoing refinement of speech output and contribute to adaptive motor learning over time. At the completion of this stage, the phonological representation has been transformed into coordinated articulatory movement, resulting in overt spoken production.

### AAC route

#### Symbol retrieval

While semantic concept retrieval, including lemma retrieval, is theorized to be common to both routes, the AAC route requires an extra level from lemma to output in order to facilitate AAC icon selection. Whereas in the spoken route, the path continues from lemma to phonological encoding to speech, on the AAC route, the path continues from lemma to a visual prototype of the concept, which then allows the user to search for and select the matching icon. We refer to this visual prototype mediating between lemma and icon as the “symbol.” This symbol differs from the lemma in that it focuses only on a subset of semantic properties (visual) for a narrow purpose (icon selection). For example, the visual properties of a “gift” in the real world vary: a gift may be square or rectangular (or another shape), with a bow, or without, while the icon for “gift” on a given AAC display will instantiate specific visual properties of “gift” (e.g., a square box with a bow on one device, a rectangular box without a bow on another). The AAC user must come up with a mental visual prototype “symbol” that abstracts away from specific visual features of “gift” before searching for a reasonable exemplar of that prototype on the device display. With practice and increased familiarity with a specific device display, this visual prototype-icon matching procedure may become increasingly automatic, so that the user skips the visual prototype “symbol” stage all together. The existence of this “symbol” stage would be indicated by increased activation in visuo-spatial processing areas compared to spoken language; evidence of automatization of this process would be decreased visuo-spatial processing in the AAC condition over the course of learning in a longitudinal design.

Rather than unfolding as strictly sequential stages, symbol retrieval, visual encoding, spatial scanning, and symbol selection are likely to operate through dynamically interacting processes. As the target symbol representation becomes activated, visual encoding of the display occurs in parallel. Primary visual cortex supports early processing of visual features, while higher order visual regions contribute to recognition of icon characteristics. At the same time, attentional control networks, including frontal eye fields and superior parietal lobule, support spatial scanning of the interface, allocating attention across the display and guiding gaze shifts toward candidate symbols (Corbetta et al., 2008; Corbetta and Shulman, 2002; Fox et al., 2006).

When multiple semantically or visually related icons are present, competition may arise at both conceptual and visual levels. Inferior frontal regions, particularly the left inferior frontal gyrus, are hypothesized to mediate controlled symbol selection by resolving conflict among competing alternatives. Importantly, visual encoding, spatial scanning, and symbol selection likely influence one another bidirectionally. The user’s ongoing visual exploration may refine the internal symbol representation, while the evolving conceptual target constrains attentional allocation across the display.

#### Hand, limb, eye motor planning and execution

Following stabilization of the target symbol through visual encoding and spatial scanning, the AAC route proceeds to motor planning and execution for body parts, such as hand, limb, or eye, depending on the user and/or system. Within the TRANSCEND Model, this stage is grounded in established models of goal-directed motor control, which describe coordinated feedforward planning, visuomotor integration, and feedback-based correction during object directed action (Scott, 2004; Shadmehr and Krakauer, 2008; Wolpert and Kawato, 1998). Once the target icon has been spatially localized, premotor cortex and supplementary motor area generate motor plans corresponding to the intended reaching, tapping, or switch activation movement. These regions encode the trajectory and sequencing of limb movements and postural control required to access the selected symbol. Posterior parietal regions, particularly superior parietal lobule and adjacent intraparietal areas, contribute to visuomotor transformation by integrating spatial information about the icon’s location with body centered motor coordinates.

Feedforward motor commands are transmitted to primary motor cortex, which executes coordinated activation of multiple muscle groups to produce goal-directed movements required for AAC access, coordinated control of the upper limb to position the hand within the workspace of the AAC device, followed by fine motor control of the fingers and wrist to accurately select target icons. These distal actions are supported by proximal joint stabilization at the shoulder and elbow and by postural control of the trunk and head that maintains a stable base for goal-directed reaching. Anticipatory postural adjustments and proximal muscle activation help align the upper limb with the display and support accurate movement trajectories. Visual cortex supports confirmation that the intended icon has been selected, while somatosensory cortex monitors limb position, joint configuration, and contact with the device surface. Discrepancies between predicted and actual movement outcomes may trigger corrective adjustments through parietal and premotor circuits, enabling real-time refinement of movement trajectories and improving the precision and efficiency of AAC access over repeated interactions. As with speech motor control, subcortical structures including basal ganglia and cerebellum contribute to timing, coordination, and error correction. At the completion of this stage, the icon (“gift” in our example) has been physically selected through coordinated limb motor execution, resulting in externalized AAC output.

For alternative access methods such as switch activation, larger proximal limb or head movements may be engaged, requiring coordinated control of trunk orientation and limb positioning to generate sufficient force and timing for switch contact. In eye gaze systems, oculomotor control networks involving the frontal eye fields and posterior parietal cortex coordinate gaze shifts toward target symbols, while head orientation and postural stability support visual alignment with the display. Simultaneously, visual and somatosensory feedback mechanisms monitor movement accuracy throughout the action.

### Dual interactive routes: the case of (re-)learning in aphasia

As seen in Figure 1A and highlighted previously, TRANSCEND models the possibility of interaction between the verbal and AAC routes during multimodal word production, defined here as simultaneous AAC-mediated and spoken output. In such a scenario, we hypothesize that AAC experience and language ability will shape both the degree to which different components of TRANSCEND are recruited and the extent of coordination between the AAC and verbal production routes. To illustrate the potential interactivity of the model and demonstrate its use, we return to our example of the individual who intends to communicate the word “gift” to describe a birthday party they attended. In this updated scenario, the individual is a person with aphasia who is learning to use their AAC device while also aiming to improve their verbal abilities.

As noted above, conveying this message begins with the Conceptual Memory Network Component, which we posit serves as a shared resource across both routes. Aphasia most commonly results from damage to lateral left cortical regions supplied by the middle cerebral artery, often leaving the neural architecture that supports episodic memory encoding and consolidation relatively spared. In parallel, neuropsychological and experimental evidence indicates that semantic knowledge is often preserved in people with post-stroke aphasia (Jefferies, 2013; Jefferies et al., 2007; Jefferies and Lambon Ralph, 2006). Thus, in our sample case, the individual would be expected to retrieve the memory of the party they attended and access the semantic representations associated with “gift,” leading to activation of regions within the Conceptual Memory Network Component.

Anomia—impaired word retrieval—is a defining characteristic of aphasia (Goodglass and Wingfield, 1997) making it unlikely that our case example would quickly access and integrate the correct lexical information needed to articulate “gift.” For someone with aphasia, potential points of breakdown during word retrieval include selecting the target lexical item among competitors, retrieving the correct phonological word form, assembling phonological codes for production, and mapping the representation onto speech motor output (Schwartz et al., 2006; Schwartz, 2014). An attempt at word retrieval would activate core frontotemporal language regions (LaCroix et al., 2021; Stefaniak et al., 2021) as well as those implicated in semantic control (Hodgson et al., 2021, 2024; Jackson, 2021) but correct versus incorrect productions may differ in activity patterns (e.g., Postman-Caucheteux et al., 2010). During this process, the MD system may also be recruited, not for core linguistic computations per se, but to support domain-general demands associated with effortful verbal production, such as maintaining the communicative goal, resolving competition among candidate responses, monitoring output, and holding partial lexical-semantic or phonological information in working memory (Diachek et al., 2020; Wehbe et al., 2021).

In the event of a retrieval failure, the individual may momentarily abandon the verbal route and turn to their AAC system. While using their AAC device, this individual will likely experience heightened demands on neural systems involved in visual search, semantic categorization, learning, and motor execution compared to experienced and proficient AAC users. During device use, the MD system will again be engaged to sustain the user’s communicative intent, resolve competition between candidate target icons, and monitor errors.

Notably, partial activation in one modality may cue processing in the other, such that written labels, icons, gestures, or partial spoken forms can mutually constrain lexical retrieval and AAC selection. In the case of this user, selection of the correct icon would rapidly re-activate lexical information, which could allow object names to become more available, facilitating overt naming (Meyer et al., 2007). Early in the AAC learning process, however, incorrect icon selection on an AAC system—for example, selecting “balloon” instead of “gift”—may reflect competition among partially learned symbol-meaning-location mappings. Although this competition should decrease as correct mappings become more automatized, simultaneous or near-simultaneous verbal and AAC productions may continue to recruit cognitive control systems to shift between modalities, coordinate multimodal output, and manage the demands of spoken language production. While there is currently little research on how speakers might switch between modes and/or use them simultaneously, this model allows for testable hypotheses in many clinical populations that can lead to further understanding of this sort of interaction.

## Functional near-infrared spectroscopy: a promising tool for investigation into the neural correlates of real-time AAC use

Testing the TRANSCEND model will require AAC researchers to adopt new tools and methodological approaches. Previous neuroimaging investigations of high-tech AAC use, while promising, were limited by the constraints of fMRI, which is expensive, requires stillness in an enclosed, noisy machine, and is difficult to implement during naturalistic communication (Dietz et al., 2018; Wilkinson et al., 2015a; Wilkinson et al., 2015b). Thus, there remains a need for more accessible and ecologically valid neuroimaging methods to study real-time AAC use in real-world contexts. As such, we propose the use of functional near-infrared spectroscopy (fNIRS) as an initial primary methodological tool to test the cortical components of our novel TRANSCEND model. Unlike fMRI, fNIRS requires only that the participant wear a non-invasive sensor cap, which can be done sitting naturally while using a high-tech AAC device. During fNIRS imaging, low powered light-emitting diodes (called sources) resting against the scalp project near-infrared light into the brain. Chromophores in deoxy- and oxyhemoglobin are sensitive to and absorb near-infrared light, resulting in a change in the amount of optical signal that returns to the head surface, as measured by photodiode detectors also on the scalp. Thus, changes in the amount of returned light reflect changes in deoxy- and oxyhemoglobin concentrations, providing an indirect measure of brain activity, like fMRI. Compared to fMRI, fNIRS is more portable, less restrictive, relatively low-cost, more robust to gross motion artifacts, and safe for individuals with implanted medical devices. fNIRS also provides higher temporal resolution than fMRI and better spatial resolution than EEG, capturing changes in blood oxygenation associated with the cognitive and motor demands of AAC use. Thus, fNIRS would enable researchers to observe brain activity during real-time communication tasks in naturalistic settings, particularly language and cortical motor processes in AAC users during their interactions with familiar communication partners, such as family members, caregivers, or speech-language pathologists. These features allow researchers to measure the neural correlates of cognitive functions such as word retrieval, working memory, attention, and executive functioning during AAC use, as well as the relationship between cortical activity and observable behaviors.

While fNIRS offers these methodological advantages, it is not without limitations. First, fNIRS signal quality can be affected by external factors such as hair density and color and scalp blood flow (Brigadoi and Cooper, 2015; Meier et al., 2025; Yücel et al., 2015, 2025). Second, its relatively shallow penetration depth precludes access to subcortical structures, meaning that fNIRS is not the ideal sole method to test TRANSCEND predictions regarding how the basal ganglia, thalamus, and brainstem contribute to feedforward and feedback motor control during AAC use or multimodal AAC-based and verbal production. While challenging, cerebellar recordings with fNIRS are feasible to obtain, including in clinical populations (Lo et al., 2025). While doing so may sacrifice the ecological validity of fNIRS-only paradigms, multimodal neuroimaging approaches, such as synchronous fNIRS and fMRI recordings (Scarapicchia et al., 2017; Yang and Wang, 2025), would allow investigators to fully test the TRANSCEND model.

Despite these constraints, the ecological validity and user-friendly nature of fNIRS make it a powerful tool for investigating the neurocognitive mechanisms of AAC-based interventions in aphasia and related populations. In particular, fNIRS is ideally suited to study language proceses due to the primarily cortical architecture of the language network; all cortical ROIs within the TRANSCEND model can be easily reached and interrogated with whole-head sensor coverage. Indeed, there is emergent demonstrated utility for its use in indexing recovery in people with aphasia, including investigation of resting state correlates of early post-stroke recovery (Meier et al., 2023), cross-sectional studies of the cognitive-linguistic processes and deficits (Gilmore et al., 2021; Li et al., 2023; Lu et al., 2025; Mohapatra and Bhattarai, 2025), and language network reorganization following non-invasive brain stimulation and/or speech-language intervention (Bunker et al., 2022; Chang et al., 2022; Gan et al., 2024; Hara et al., 2017; Wang et al., 2025; Xu et al., 2025). These studies provide a solid foundation for exploring the neural correlates of AAC intervention in aphasia, including potential language recovery associated with AAC use.

Building on its validated use in aphasia research, fNIRS offers a promising methodological foundation for investigating AAC intervention more broadly, beyond aphasia. Individuals who use AAC systems are commonly evaluated on multiple communicative competencies including linguistic, operational, strategic, social, and psychosocial domains (Light and McNaughton, 2014). While fNIRS is not designed to capture all of these dimensions, it has been successfully used to study core neurocognitive processes that underline several of them. For example, researchers have used fNIRS to examine skills relevant to operational competence, such as motor planning (Alves Heinze et al., 2019; Polskaia et al., 2023), and to detect changes in cortical activation following practice of visuomotor coordination tasks, such as joystick-based cursor control (Tinga et al., 2021). While these studies did not involve AAC, they demonstrate that fNIRS is sensitive to practice related changes in motor and executive system engagement during complex, goal directed tasks. Collectively, this methodological evidence supports the application of fNIRS to AAC research, where successful device use similarly requires coordinated language, visuospatial, motor, and executive processes.

Furthermore, our group has conducted preliminary research using fNIRS to explore AAC use more directly. In order to investigate how practice schedules rooted in motor learning theory affect AAC learning and brain activity, we recruited four adult participants who completed either blocked or random practice to learn target symbols on a high-tech AAC system while accuracy and fNIRS data were collected. Initial results showed that random practice was associated with higher accuracy and individual-level changes in cortical activation over time. While limited in sample size, these findings provide early support for the feasibility of using fNIRS to study the neurocognitive processes involved in AAC learning (Edwards et al., 2025). Taken with the previous literature cited above, this preliminary research provides further evidence that fNIRS is a feasible approach to mapping neural activity associated with real-time AAC use and examining how the relevant systems are engaged and potentially reorganized during intervention in individuals with aphasia.

Our team is also currently collecting preliminary data from an fNIRS study designed specifically to investigate aspects of the TRANSCEND framework. Our pilot study involves a one-session training in which participants learn to produce words using an AAC device, followed by an experimental task in which they undergo fNIRS scanning while producing words in alternating AAC and spoken word conditions. The TRANSCEND model predicts that for the AAC condition compared to the spoken condition, there should be increased activation in areas associated with cognitive control, dorsal motor areas associated with the limb/hands, and visual cortex areas. Conversely, the spoken condition is predicted to show more activation than the AAC condition in regions associated with phonological processing, as well as in ventral motor areas associated with the face. Since the semantic network is hypothesized to be active in both conditions, no difference is expected across conditions. Our pilot data on healthy participants conforms to these predictions; the AAC condition showed higher activation in lateral prefrontal areas associated with cognitive control and decreased activation in temporoparietal areas associated with phonological processing. While our current equipment limitations did not allow for fNIRS coverage of the motor and occipital regions, these initial results are promising for future studies with a larger fNIRS montage. This kind of study can also be expanded from an initial convenience sample of healthy participants completing a one-session AAC training to a more involved longitudinal study of clinical populations undergoing an AAC-based intervention with fNIRS probes at multiple time points, in order to investigate neurological changes over long-term training, with comparison to the model.

## Establishing a neuroimaging paradigm for AAC research

The field of AAC has made remarkable strides in improving communicative outcomes, yet the neurological mechanisms underlying its success and potential co-occurring language recovery in neurogenic populations remain underexplored. In this paper, we have argued that AAC should be re-theorized not only as compensatory support with observable behavioral outcomes, but also as a neurocognitive interface, one that interacts dynamically with core cognitive domains and holds the potential to drive neuroplastic change. To this end, we have proposed a neurocognitive framework, the TRANSCEND model, in which to ground a body of empirical research that tests this claim. Specifically, empirical research is necessary to differentiate between brain changes driven by the therapeutic effects of high-technology AAC interventions on speech and language outcomes in people with communication disorders (e.g., aphasia) and those reflecting the cognitive, linguistic, and motor demands inherent to AAC device use. A clear neurocognitive framework integrating verbal and AAC-based output routes, rooted in previously-establish component process models, will allow for grounded empirical research that teases apart language-general and modality-specific brain activation patterns in this way; we offer the TRANSCEND model as one such framework.

Building on a foundation of previous fNIRS studies, we propose a research agenda using fNIRS to explore neural activation patterns during real-time high-tech AAC use, including longitudinal studies that track changes in brain activity and language function during high-tech AAC intervention, guided by our TRANSCEND model. This approach has the potential to reveal neural correlates of high-tech AAC linguistic production, and, further, how AAC use contributes to recovery and neural plasticity in individuals with aphasia. This groundwork layer of empirical research can also be used in the future for other empirical applications, such as critically evaluating the growing integration of artificial intelligence into commercial AAC technologies, which often outpaces empirical validation, and, ultimately, for theoretical applications, such as informing and refining neurocognitive models of AAC-supported communication more broadly, including interaction with a communication partner and integration of low-tech strategies like gesture.

Furthermore, a deeper understanding of the neurocognitive underpinnings of high-tech AAC use could also refine clinical practice, inform intervention design, and reshape our assumptions about how individuals with severe communication disorders process and produce language when using aided communication devices in addition to natural speech; specifically, by enabling clinicians to identify specific points of breakdown within AAC language processing, allowing intervention to target disrupted processes rather than abandoning AAC altogether. By shifting the focus from device use to underlying cognitive mechanisms, this approach may also help dispel persistent myths that AAC hinders speech-language development or recovery, or that it is ineffective.

As Dietz et al. (2020) argue, high-tech AAC should not be viewed solely as a replacement for speech but as a “dual purpose treatment” that enhances both communication and language recovery (p. 16). The time has come to move beyond asking whether AAC works and begin investigating how it works at a neural level. While AAC is not universally adopted and outcomes vary across individuals, such variability highlights the need to understand the underlying neurocognitive processes that support successful implementation. AAC can be effective, but its success depends on how well it is individually titrated and explicitly trained to align with a user’s linguistic, motor, visuospatial, and executive capacities. Embracing this challenge will refine our intervention strategies, deepen our understanding of brain behavior relationships, and ultimately empower more individuals to access and express their full communicative potential.

## Data Availability

The original contributions presented in the study are included in the article/supplementary material, further inquiries can be directed to the corresponding author.

## References

[ref1] Alam N. Munjal S. Panda N. K. Kumar R. Gupta S. (2023). Efficacy of Jellow app as an adjunct to stimulation therapy in improvement in language and quality of life in patients with chronic Broca’s aphasia. Disabil. Rehabil. Assist. Technol. 18, 596–602. doi: 10.1080/17483107.2021.1892844, 33689529

[ref2] Alves Heinze R. Vanzella P. Zimeo Morais G. A. Sato J. R. (2019). Hand motor learning in a musical context and prefrontal cortex hemodynamic response: a functional near-infrared spectroscopy (fNIRS) study. Cogn. Process. 20, 507–513. doi: 10.1007/s10339-019-00925-y, 31385142

[ref3] Alzrayer N. M. Aldabas R. Alhossein A. Alharthi H. (2021). Naturalistic teaching approach to develop spontaneous vocalizations and augmented communication in children with autism spectrum disorder. Augment. Altern. Commun. 37, 14–24. doi: 10.1080/07434618.2021.1881825, 33825612

[ref4] Beukelman D. R. Light J. C. (2020). Augmentative & alternative communication: Supporting children and adults with complex communication needs (Fifth edition). Baltimore: Brookes Publishing Co. (Accessed April 7, 2026).

[ref5] Binder J. R. Desai R. H. (2011). The neurobiology of semantic memory. Trends Cogn. Sci. 15, 527–536. doi: 10.1016/j.tics.2011.10.001, 22001867 PMC3350748

[ref6] Bohland J. W. Bullock D. Guenther F. H. (2010). Neural representations and mechanisms for the performance of simple speech sequences. J. Cogn. Neurosci. 22, 1504–1529. doi: 10.1162/jocn.2009.21306, 19583476 PMC2937837

[ref7] Braver T. S. Kizhner A. Tang R. Freund M. C. Etzel J. A. (2021). The dual mechanisms of cognitive control project. J. Cogn. Neurosci. 1–26, 1–26. doi: 10.1162/jocn_a_01768, 34407191 PMC10069323

[ref8] Brigadoi S. Cooper R. J. (2015). How short is short? Optimum source–detector distance for short-separation channels in functional near-infrared spectroscopy. Neurophotonics 2:025005. doi: 10.1117/1.NPh.2.2.025005, 26158009 PMC4478880

[ref9] Bunker L. Kim H. Meier E. Hillis A. (2022). Variable picture naming activation patterns in post-stroke aphasia: a functional near-infrared spectroscopy (fNIRS) case series investigation. (S17.006). Neurology 98:3052. doi: 10.1212/WNL.98.18_supplement.3052

[ref10] Chang W. K. Park J. Lee J.-Y. Cho S. Lee J. Kim W.-S. . (2022). Functional network changes after high-frequency rTMS over the Most activated speech-related area combined with speech therapy in chronic stroke with non-fluent aphasia. Front. Neurol. 13:690048. doi: 10.3389/fneur.2022.690048, 35222235 PMC8866644

[ref11] Chen S. H. K. Hill K. McNeil M. R. Wang E.-H. Zhou L. (2026a). A preliminary study investigating a language intervention for people with aphasia comparing human versus AAC-generated speech feedback. Disabil. Rehabil. Assist. Technol. 1–17. doi: 10.1080/17483107.2026.2626464, 41693491

[ref12] Chen S. H. K. O’Donnell-Nolin J. Morgan M. (2026b). Person-centered augmentative and alternative communication with verb network strengthening training: a case study in Broca’s aphasia. Aphasiology. 1–22. doi: 10.1080/02687038.2026.2618699, 37339054

[ref13] Cieslik E. C. Mueller V. I. Eickhoff C. R. Langner R. Eickhoff S. B. (2015). Three key regions for supervisory attentional control: evidence from neuroimaging meta-analyses. Neurosci. Biobehav. Rev. 48, 22–34. doi: 10.1016/j.neubiorev.2014.11.003, 25446951 PMC4272620

[ref14] Corbetta M. Patel G. Shulman G. L. (2008). The reorienting system of the human brain: from environment to theory of mind. Neuron 58, 306–324. doi: 10.1016/j.neuron.2008.04.017, 18466742 PMC2441869

[ref15] Corbetta M. Shulman G. L. (2002). Control of goal-directed and stimulus-driven attention in the brain. Nat. Rev. Neurosci. 3, 201–215. doi: 10.1038/nrn755, 11994752

[ref16] Crittenden B. M. Mitchell D. J. Duncan J. (2016). Task encoding across the multiple demand cortex is consistent with a frontoparietal and Cingulo-opercular dual networks distinction. J. Neurosci. 36, 6147–6155. doi: 10.1523/JNEUROSCI.4590-15.2016, 27277793 PMC4899522

[ref17] Dell G. S. O’Seaghdha P. G. (1992). Stages of lexical access in language production. Cognition 42, 287–314. doi: 10.1016/0010-0277(92)90046-K, 1582160

[ref18] Dell G. S. Schwartz M. F. Martin N. Saffran E. M. Gagnon D. A. (1997). Lexical access in aphasic and nonaphasic speakers. Psychol. Rev. 104, 801–838. doi: 10.1037/0033-295X.104.4.801, 9337631

[ref19] Diachek E. Blank I. Siegelman M. Affourtit J. Fedorenko E. (2020). The domain-general multiple demand (MD) network does not support Core aspects of language comprehension: a large-scale fMRI investigation. J. Neurosci. 40, 4536–4550. doi: 10.1523/JNEUROSCI.2036-19.2020, 32317387 PMC7275862

[ref20] Dietz A. R. Vannest J. Collier J. Maloney T. Altaye M. Szaflarski J. . (2014). AAC Revolutionizes Aphasia Therapy: Changes in Cortical Plasticity and Spoken Language Production. Clinical Aphasiology Paper. Available online at: http://aphasiology.pitt.edu/archive/00002557/ (Accessed April 7, 2026).

[ref21] Dietz A. Vannest J. Maloney T. Altaye M. Holland S. Szaflarski J. P. (2018). The feasibility of improving discourse in people with aphasia through AAC: clinical and functional MRI correlates. Aphasiology 32, 693–719. doi: 10.1080/02687038.2018.1447641, 32999522 PMC7523709

[ref22] Dietz A. Wallace S. E. Weissling K. (2020). Revisiting the role of augmentative and alternative communication in aphasia rehabilitation. Am. J. Speech Lang. Pathol. 29, 909–913. doi: 10.1044/2019_AJSLP-19-00041, 32109137 PMC7842873

[ref23] Dosenbach N. U. F. Fair D. A. Cohen A. L. Schlaggar B. L. Petersen S. E. (2008). A dual-networks architecture of top-down control. Trends Cogn. Sci. 12, 99–105. doi: 10.1016/j.tics.2008.01.001, 18262825 PMC3632449

[ref24] Dosenbach N. U. F. Fair D. A. Miezin F. M. Cohen A. L. Wenger K. K. Dosenbach R. A. T. . (2007). Distinct brain networks for adaptive and stable task control in humans. Proc. Natl. Acad. Sci. 104, 11073–11078. doi: 10.1073/pnas.0704320104, 17576922 PMC1904171

[ref25] Duncan J. (2010). The multiple-demand (MD) system of the primate brain: mental programs for intelligent behaviour. Trends Cogn. Sci. 14, 172–179. doi: 10.1016/j.tics.2010.01.004, 20171926

[ref26] Edwards K. Shine K. Tockman H. Liu H. T. Lavallee J. Chen K. (2025). Exploring Practice Schedules in AAC Vocabulary Learning: A Pilot fNIRS Study on Cortical Activation. American Speech-Language-Hearing Association Convention. Washington, D.C.

[ref27] Fedorenko E. Behr M. K. Kanwisher N. (2011). Functional specificity for high-level linguistic processing in the human brain. Proc. Natl. Acad. Sci. 108, 16428–16433. doi: 10.1073/pnas.1112937108, 21885736 PMC3182706

[ref28] Fedorenko E. Duncan J. Kanwisher N. (2013). Broad domain generality in focal regions of frontal and parietal cortex. Proc. Natl. Acad. Sci. 110, 16616–16621. doi: 10.1073/pnas.1315235110, 24062451 PMC3799302

[ref29] Fedorenko E. Ivanova A. A. Regev T. I. (2024). The language network as a natural kind within the broader landscape of the human brain. Nat. Rev. Neurosci. 25, 289–312. doi: 10.1038/s41583-024-00802-4, 38609551 PMC13222024

[ref30] Fox M. D. Corbetta M. Snyder A. Z. Vincent J. L. Raichle M. E. (2006). Spontaneous neuronal activity distinguishes human dorsal and ventral attention systems. Proc. Natl. Acad. Sci. 103, 10046–10051. doi: 10.1073/pnas.0604187103, 16788060 PMC1480402

[ref31] Fried-Oken M. Beukelman D. R. Hux K. (2012). Current and future AAC research considerations for adults with acquired cognitive and communication impairments. Assist. Technol. 24, 56–66. doi: 10.1080/10400435.2011.648713, 22590800 PMC3760684

[ref32] Fried-Oken M. Fox L. Rau M. T. Tullman J. Baker G. Hindal M. . (2006). Purposes of AAC device use for persons with ALS as reported by caregivers. Augment. Altern. Commun. 22, 209–221. doi: 10.1080/07434610600650276, 17114164

[ref33] Gan L. Huang L. Zhang Y. Yang X. Li L. Meng L. . (2024). Effects of low-frequency rTMS combined with speech and language therapy on Broca’s aphasia in subacute stroke patients. Front. Neurol. 15:1473254. doi: 10.3389/fneur.2024.1473254, 39539660 PMC11557360

[ref34] Gilmore N. Yücel M. A. Li X. Boas D. A. Kiran S. (2021). Investigating language and domain-general processing in neurotypicals and individuals with aphasia—a functional near-infrared spectroscopy pilot study. Front. Hum. Neurosci. 15:728151. doi: 10.3389/fnhum.2021.728151, 34602997 PMC8484538

[ref35] Goodglass H. Wingfield A. (1997). Anomia: Neuroanatomical and Cognitive correlates. New York: Academic Press.

[ref36] Hara T. Abo M. Kakita K. Mori Y. Yoshida M. Sasaki N. (2017). The effect of selective transcranial magnetic stimulation with functional near-infrared spectroscopy and intensive speech therapy on individuals with post-stroke aphasia. Eur. Neurol. 77, 186–194. doi: 10.1159/000457901, 28161706

[ref37] Hodgson V. J. Lambon Ralph M. A. Jackson R. L. (2021). Multiple dimensions underlying the functional organization of the language network. NeuroImage 241:118444. doi: 10.1016/j.neuroimage.2021.118444, 34343627 PMC8456749

[ref38] Hodgson V. J. Ralph M. A. L. Jackson R. L. (2024). Disentangling the neural correlates of semantic and domain-general control: the roles of stimulus domain and task process. Imaging Neurosci. 2:imag–2–00092. doi: 10.1162/imag_a_00092, 40800362 PMC12224409

[ref39] Holyfield C. Drager K. D. R. Kremkow J. M. D. Light J. (2017). Systematic review of AAC intervention research for adolescents and adults with autism spectrum disorder. Augment. Altern. Commun. 33, 201–212. doi: 10.1080/07434618.2017.1370495, 28884601

[ref40] Huang L. Yan J. Chen S.-H. K. (2025). Combining low-technology augmentative and alternative communication with regular aphasia treatment: interim findings of a hospital-based RCT in post-stroke patients. Aphasiology 39, 1144–1163. doi: 10.1080/02687038.2024.2406592

[ref41] Jackson R. L. (2021). The neural correlates of semantic control revisited. NeuroImage 224:117444. doi: 10.1016/j.neuroimage.2020.117444, 33059049 PMC7779562

[ref42] Jefferies E. (2013). The neural basis of semantic cognition: converging evidence from neuropsychology, neuroimaging and TMS. Cortex 49, 611–625. doi: 10.1016/j.cortex.2012.10.008, 23260615

[ref43] Jefferies E. Baker S. S. Doran M. Ralph M. A. L. (2007). Refractory effects in stroke aphasia: a consequence of poor semantic control. Neuropsychologia 45, 1065–1079. doi: 10.1016/j.neuropsychologia.2006.09.009, 17074373

[ref44] Jefferies E. Lambon Ralph M. A. (2006). Semantic impairment in stroke aphasia versus semantic dementia: a case-series comparison. Brain 129, 2132–2147. doi: 10.1093/brain/awl153, 16815878

[ref45] Kraat A. W. (1990). Augmentative and alternative communication: does it have a future in aphasia rehabilitation? Aphasiology 4, 321–338. doi: 10.1080/02687039008249086

[ref46] LaCroix A. N. James E. Rogalsky C. (2021). Neural resources supporting language production vs. comprehension in chronic post-stroke aphasia: a meta-analysis using activation likelihood estimates. Front. Hum. Neurosci. 15:680933. doi: 10.3389/fnhum.2021.680933, 34759804 PMC8572938

[ref47] Lambon Ralph M. A. L. Jefferies E. Patterson K. Rogers T. T. (2017). The neural and computational bases of semantic cognition. Nat. Rev. Neurosci. 18, 42–55. doi: 10.1038/nrn.2016.150, 27881854

[ref48] Levelt W. J. M. Roelofs A. Meyer A. S. (1999). A theory of lexical access in speech production. Behav. Brain Sci. 22, 1–38. doi: 10.1017/S0140525X99001776, 11301520

[ref49] Li H. Liu J. Tian S. Fan S. Wang T. Qian H. . (2023). Language reorganization patterns in global aphasia–evidence from fNIRS. Front. Neurol. 13:1025384. doi: 10.3389/fneur.2022.1025384, 36686505 PMC9853054

[ref50] Light J. McNaughton D. (2014). Communicative competence for individuals who require augmentative and alternative communication: a new definition for a new era of communication? Augment. Altern. Commun. 30, 1–18. doi: 10.3109/07434618.2014.885080, 30952185

[ref51] Lo Y. L. Chia G. T. H. Chen Y. T. C. Tan E. K. (2025). Near-infrared spectroscopy of the cerebellum in motor activation tasks. Sci. Rep. 15:21608. doi: 10.1038/s41598-025-04678-x, 40592959 PMC12216116

[ref52] Lu C. Wang M. Zhan L. Lu M. (2025). Unveiling cognitive interference: fNIRS insights into poststroke aphasia during Stroop tasks. Neural Plast. 2025:1456201. doi: 10.1155/np/1456201, 40201621 PMC11976049

[ref53] MacGregor L. J. Gilbert R. A. Balewski Z. Mitchell D. J. Erzinçlioğlu S. W. Rodd J. M. . (2022). Causal contributions of the domain-general (multiple demand) and the language-selective brain networks to perceptual and semantic challenges in speech comprehension. Neurobiol. Lang. 3, 665–698. doi: 10.1162/nol_a_00081, 36742011 PMC9893226

[ref54] May A. A. Dada S. Murray J. (2024). Identifying components of a person-centered augmentative and alternative communication intervention for people with dementia: opinions of an international expert panel. Am. J. Speech Lang. Pathol. 33, 2067–2082. doi: 10.1044/2024_AJSLP-23-00317, 38901000

[ref55] Meier E. L. Bunker L. D. Kim H. Durfee A. Z. Tilton-Bolowsky V. Neal V. . (2025). The effects of protocol factors and participant characteristics on functional near-infrared spectroscopy data quality after stroke. NeuroImage: Reports 5:100276. doi: 10.1016/j.ynirp.2025.100276, 41050942 PMC12489784

[ref56] Meier E. L. Bunker L. D. Kim H. Hillis A. E. (2023). Resting-state connectivity in acute and subacute poststroke aphasia: a functional near-infrared spectroscopy pilot study. Brain Connect. 13, 441–452. doi: 10.1089/brain.2022.0065, 37097208 PMC10618818

[ref57] Menon V. Uddin L. Q. (2010). Saliency, switching, attention and control: a network model of insula function. Brain Struct. Funct. 214, 655–667. doi: 10.1007/s00429-010-0262-0, 20512370 PMC2899886

[ref58] Meyer A. S. Belke E. Telling A. L. Humphreys G. W. (2007). Early activation of object names in visual search. Psychon. Bull. Rev. 14, 710–716. doi: 10.3758/BF03196826, 17972738

[ref59] Miller E. K. Cohen J. D. (2001). An integrative theory of prefrontal cortex function. Annu. Rev. Neurosci. 24, 167–202. doi: 10.1146/annurev.neuro.24.1.167, 11283309

[ref60] Mirman D. Landrigan J.-F. Britt A. E. (2017). Taxonomic and thematic semantic systems. Psychol. Bull. 143, 499–520. doi: 10.1037/bul0000092, 28333494 PMC5393928

[ref61] Mohapatra B. Bhattarai B. (2025). Prefrontal cortex activation and hemispheric lateralization during working memory load processing in individuals with aphasia: an fNIRS study. Aphasiology. 1–36. doi: 10.1080/02687038.2025.2586583, 37339054

[ref62] Noonan K. A. Jefferies E. Visser M. Lambon Ralph M. A. (2013). Going beyond inferior prefrontal involvement in semantic control: evidence for the additional contribution of dorsal angular gyrus and posterior middle temporal cortex. J. Cogn. Neurosci. 25, 1824–1850. doi: 10.1162/jocn_a_00442, 23859646

[ref63] Polskaia N. St-Amant G. Fraser S. Lajoie Y. (2023). Involvement of the prefrontal cortex in motor sequence learning: a functional near-infrared spectroscopy (fNIRS) study. Brain Cogn. 166:105940. doi: 10.1016/j.bandc.2022.105940 36621187

[ref64] Pope L. Light J. Laubscher E. (2025). The effect of naturalistic developmental behavioral interventions and aided AAC on the language development of children on the autism spectrum with minimal speech: a systematic review and meta-analysis. J. Autism Dev. Disord. 55, 3078–3099. doi: 10.1007/s10803-024-06382-7, 38848009 PMC12208088

[ref65] Postman-Caucheteux W. A. Birn R. M. Pursley R. H. Butman J. A. Solomon J. M. Picchioni D. . (2010). Single-trial fMRI shows contralesional activity linked to overt naming errors in chronic aphasic patients. J. Cogn. Neurosci. 22, 1299–1318. doi: 10.1162/jocn.2009.21261, 19413476 PMC4778722

[ref66] Pousada García T. Pereira Loureiro J. Groba González B. Nieto Riveiro L. Pazos Sierra A. (2011). The use of computers and augmentative and alternative communication devices by children and young with cerebral palsy. Assist. Technol. 23, 135–149. doi: 10.1080/10400435.2011.588988

[ref67] Pulvermüller F. Berthier M. L. (2008). Aphasia therapy on a neuroscience basis. Aphasiology 22, 563–599. doi: 10.1080/02687030701612213, 18923644 PMC2557073

[ref69] Scarapicchia V. Brown C. Mayo C. Gawryluk J. R. (2017). Functional magnetic resonance imaging and functional near-infrared spectroscopy: insights from combined recording studies. Front. Hum. Neurosci. 11:419. doi: 10.3389/fnhum.2017.00419, 28867998 PMC5563305

[ref70] Schlosser R. W. Koul R. K. (2015). Speech output technologies in interventions for individuals with autism spectrum disorders: a scoping review. Augment. Altern. Commun. 31, 285–309. doi: 10.3109/07434618.2015.1063689, 26170252

[ref71] Schwartz M. F. (2014). Theoretical analysis of word production deficits in adult aphasia. Philos. Trans. R. Soc. B Biol. Sci. 369:20120390. doi: 10.1098/rstb.2012.0390, 24324234 PMC3866420

[ref72] Schwartz M. Dell G. Martin N. Gahl S. Sobel P. (2006). A case-series test of the interactive two-step model of lexical access: evidence from picture naming☆. J. Mem. Lang. 54, 228–264. doi: 10.1016/j.jml.2005.10.001 PMC2981040 21085621

[ref73] Scott S. H. (2004). Optimal feedback control and the neural basis of volitional motor control. Nat. Rev. Neurosci. 5, 532–545. doi: 10.1038/nrn1427, 15208695

[ref74] Shadmehr R. Krakauer J. W. (2008). A computational neuroanatomy for motor control. Exp. Brain Res. 185, 359–381. doi: 10.1007/s00221-008-1280-5, 18251019 PMC2553854

[ref76] Stefaniak J. D. Alyahya R. S. W. Lambon Ralph M. A. (2021). Language networks in aphasia and health: a 1000 participant activation likelihood estimation meta-analysis. NeuroImage 233:117960. doi: 10.1016/j.neuroimage.2021.117960, 33744459

[ref77] Tinga A. M. Clim M.-A. De Back T. T. Louwerse M. M. (2021). Measures of prefrontal functional near-infrared spectroscopy in visuomotor learning. Exp. Brain Res. 239, 1061–1072. doi: 10.1007/s00221-021-06039-2, 33528598 PMC8068645

[ref78] Tourville J. A. Guenther F. H. (2011). The DIVA model: a neural theory of speech acquisition and production. Lang. Cogn. Proc. 26, 952–981. doi: 10.1080/01690960903498424, 23667281 PMC3650855

[ref79] Uddin L. Q. (2015). Salience processing and insular cortical function and dysfunction. Nat. Rev. Neurosci. 16, 55–61. doi: 10.1038/nrn3857, 25406711

[ref80] Wang M.-H. Gao J. Dou W.-W. Yin L. Z. Sun Y.-T. Jiang Z.-L. . (2025). Cerebral reorganization patterns predicting language recovery in individuals with post-stroke anomia: evidence from functional near-infrared spectroscopy. J. Neurolinguistics 76:101280. doi: 10.1016/j.jneuroling.2025.101280

[ref81] Wehbe L. Blank I. A. Shain C. Futrell R. Levy R. Von Der Malsburg T. . (2021). Incremental language comprehension difficulty predicts activity in the language network but not the multiple demand network. Cereb. Cortex 31, 4006–4023. doi: 10.1093/cercor/bhab065, 33895807 PMC8328211

[ref82] Weissling K. Prentice C. (2010). The timing of remediation and compensation rehabilitation programs for individuals with acquired brain injuries: opening the conversation. Perspect. Augment. Altern. Commun. 19, 87–96. doi: 10.1044/aac19.3.87

[ref83] Wilkinson K. M. Dennis N. A. Webb C. E. Therrien M. Stradtman M. Farmer J. . (2015a). Neural activity associated with visual search for line drawings on AAC displays: an exploration of the use of fMRI. Augment. Altern. Commun. 31, 310–324. doi: 10.3109/07434618.2015.1100215, 26517757

[ref84] Wilkinson K. M. Stutzman A. Seisler A. (2015b). N400 brain responses to spoken phrases paired with photographs of scenes: implications for visual scene displays in AAC systems. Augment. Altern. Commun. 31, 51–62. doi: 10.3109/07434618.2014.965342, 25521434

[ref85] Wolpert D. M. Kawato M. (1998). Multiple paired forward and inverse models for motor control. Neural Netw. 11, 1317–1329. doi: 10.1016/S0893-6080(98)00066-5, 12662752

[ref86] Xu X. Chen S. Li Z. Zhang L. Pan T. Long S. . (2025). Efficacy of cerebellar cathodal transcranial direct current stimulation for post-stroke aphasia: a randomized controlled trial. Clin. Neurophysiol. Pract. 10, 464–473. doi: 10.1016/j.cnp.2025.10.001, 41142047 PMC12550330

[ref87] Yang L. Wang Z. (2025). Applications and advances of combined fMRI-fNIRs techniques in brain functional research. Front. Neurol. 16:1542075. doi: 10.3389/fneur.2025.1542075, 40170894 PMC11958174

[ref88] Yücel M. A. Anderson J. E. Rogers D. Hajirahimi P. Farzam P. Gao Y. . (2025). Quantifying the impact of hair and skin characteristics on fNIRS signal quality for enhanced inclusivity. Nat. Hum. Behav. 9, 2651–2668. doi: 10.1038/s41562-025-02274-7 40897802 PMC13264803

[ref89] Yücel M. A. Selb J. Aasted C. M. Petkov M. P. Becerra L. Borsook D. . (2015). Short separation regression improves statistical significance and better localizes the hemodynamic response obtained by near-infrared spectroscopy for tasks with differing autonomic responses. Neurophotonics 2:035005. doi: 10.1117/1.NPh.2.3.035005, 26835480 PMC4717232

